# Data Resources and Computational Methods for Lactylation Site Prediction: A Mini-Review

**DOI:** 10.3390/ijms27114860

**Published:** 2026-05-28

**Authors:** Cong Wang, Ye Pan, Yunlong Wu, Xiaolin Wu

**Affiliations:** 1 Data and Informatization Department, Jiangsu University, Zhenjiang 212013, China; 2 Public Experiment & Service Center, Jiangsu University, Zhenjiang 212013, China

**Keywords:** lysine lactylation, post-translational modification, computational prediction, machine learning, deep learning

## Abstract

Lysine lactylation (Kla), a novel post-translational modification (PTM) discovered in 2019, establishes a critical link between cellular metabolism and epigenetic regulation. A growing number of studies have reported that it is involved in several physiological and pathological processes. Traditional experimental methods for identifying Kla sites are time-consuming and labor-intensive; in contrast, computational prediction models offer efficient and systematic alternatives for high-throughput screening of potential modification sites. In this review, we summarize computational methods and data resources used for Kla site prediction. The biological roles of Kla in major human diseases, such as cancers, cardiovascular diseases, and neurological diseases, were summarized. Furthermore, a summary and comprehensive overview of seven Kla site prediction models is presented, covering dataset construction, methodological principles, and evaluation methods. Finally, the challenges and future trends in Kla prediction have been discussed.

## 1. Introduction

**Figure 1:**
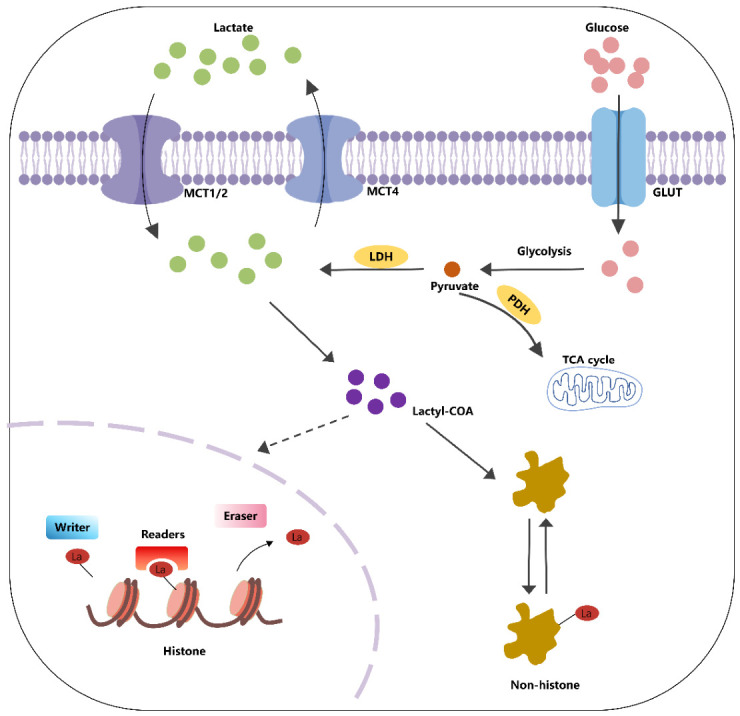
The mechanisms of lactylation generation. The main sources of lactate include glycolysis or transport via the MCT1/2 transporter proteins. Lactyl-coenzyme A (Lactyl-CoA) is produced from lactate and acts as a key substrate for the lactylation of both histones and non-histone proteins. The dynamic regulation of histone lactylation is governed by three functional classes of proteins: “writers” that catalyze the attachment of lactyl groups, “erasers” that reverse this modification, and “readers” that specifically recognize and bind to lactylated sites. Through their coordinated actions, these enzymes modulate the placement and removal of lactyl marks on histone lysine residues. In the case of non-histone proteins, lactylation affects their biological activity by reshaping the three-dimensional conformation of the target molecules. MCT, monocarboxylate transporter; GLUT, glucose transporter; LDH, lactate dehydrogenase; PDH, pyruvate dehydrogenase; TCA cycle, tricarboxylic acid cycle. Created using Adobe Illustrator 2024.

Lactate was long considered merely a metabolic waste product of glycolysis under anaerobic conditions [1]. However, recent studies have revealed that lactate functions as a key signaling molecule involved in signal transduction, immune regulation, and epigenetic modification [2]. In 2019, Zhang et al. first reported that lactate can modify lysine residues on histones, forming a novel epigenetic mark known as histone lactylation (Kla). They confirmed that this modification is an active enzyme-catalyzed process mediated by lactyl-CoA [3]. In 2020, Gaffney et al. reported that non-enzymatic lactylation of glycolytic enzymes (non-histone proteins) occurs via lactoylglutathione. This modification exerts negative feedback regulation on the glycolytic pathway (Figure 1) [4].

Histone lactylation regulates gene transcription by altering chromatin structure, modulating transcription factor binding activity, and dynamically influencing the accessibility of gene promoter regions [5,6,7,8]. Histone lactylation predominantly occurs on the four core histones (H2A, H2B, H3, and H4), with H3K18la and H4K12la being the most extensively studied sites [3,9]. H3K18la has been shown to promote gastric cancer cell proliferation and induce immune escape in non-small cell lung cancer, leading to poor prognosis and immunotherapy resistance [10,11]. H4K12la drives psoriasis progression by enhancing IL-17A-mediated keratinocyte proliferation. Non-histone protein lactylation is widely distributed among enzymes, transcription factors, and DNA damage repair proteins, regulating protein activity, stability, and interaction networks [4,12]. For instance, ABCF1 lactylation at K430 forms a closed regulatory loop with HIF1A to promote hepatocellular carcinoma progression [13], while Serpina3k lactylation at K351 enhances protein stability and protects cardiomyocytes from ischemia–reperfusion injury [14].

Traditional experimental methods (such as mass spectrometry and immunoblotting) for identifying Kla sites are limited by low throughput and high cost [15,16]. In contrast, computational prediction models enable efficient and systematic screening of potential Kla sites by integrating sequence and structural features of proteins [17,18,19,20,21,22]. In this review, we comprehensively summarize currently available computational methods for Kla site prediction. We focus on methodological principles, dataset construction, and evaluation methods.

## 2. Biological Roles of Kla in Physiological and Pathological Processes

Kla is closely related to disease development and progression. Three common types of diseases are described below, including cancer, cardiovascular diseases (CVDs), and neurological disorders [1,23,24,25,26,27].

### 2.1. Kla and Cancer

The Warburg effect, a hallmark of cancer metabolism, is characterized by enhanced glycolysis even under aerobic conditions, leading to excessive lactate accumulation in the tumor microenvironment [28,29,30]. Kla couples cancer metabolism with gene transcription, playing a crucial role in tumorigenesis, progression, and therapy resistance. Several studies have focused on Kla research in various types of cancer. For example, in breast cancer, lactate-induced histone lactylation regulates the expression of epithelial–mesenchymal transition (EMT)-related genes, promoting tumor invasion and metastasis [31,32]. In colorectal cancer, hypoxia-induced lactylation of β-catenin enhances its stability and activates the Wnt signaling pathway, promoting cell proliferation and stemness maintenance [33]. GPR37 activates the Hippo pathway to enhance glycolysis and histone lactylation, upregulating CXCL1 and CXCL5 expression and thereby mediating colorectal cancer liver metastasis [34]. It has been demonstrated that Kla is also closely associated with drug resistance in colorectal cancer. Bevacizumab-resistant patients often present with elevated levels of histone lactylation (specifically H3K18la). H3K18la promotes transcription of the autophagy enhancer gene RUBCNL/Pacer, strengthens its interaction with BECN1, and facilitates autophagosome maturation, thereby helping cancer cells survive in the hypoxic environment and develop drug resistance. Although Kla generally promotes a pro-tumor microenvironment, it can also act as a “double-edged sword” under certain conditions. In acute myeloid leukemia, STAT5-mediated glycolysis upregulation increases lactate levels, which promote histone lactylation and subsequent PD-L1 expression, thereby suppressing anti-tumor immunity [35,36,37].

Currently, accumulating evidence indicates that Kla is closely associated with oncogenic processes in multiple cancers, yet considerable controversies and knowledge gaps remain; it is still unclear whether Kla uniformly promotes tumor progression or exerts context-dependent tumor-suppressive effects. No bona fide specific lactylation “erasers” have been identified in the field, which hampers precise mechanistic dissection and targeted intervention. Given these unresolved issues, as well as the technical difficulties in detecting lactylation with high specificity, computational prediction becomes critically important.

### 2.2. Kla and CVD

The prevalence of cardiovascular disease continues to increase globally. Studies have shown that Kla plays a pivotal role in the pathogenesis of CVDs, including atherosclerosis (AS) [38,39], myocardial infarction (MI) [40], and ischemia–reperfusion injury (IRI) [41,42]. However, Kla exhibits strong context-dependent effects across different cardiovascular cell types. In vascular endothelial cells, lipid peroxidation induces aberrant aerobic glycolysis, leading to lactate accumulation and subsequent upregulation of H3K18la. The histone chaperone ASF1A cooperates with the acetyltransferase P300 to synergistically regulate H3K18la, thereby activating the endothelial–mesenchymal transition (EndMT) transcription factor SNAI1 and inducing endothelial dysfunction, which promotes AS progression [43]. In cardiomyocytes, the lactylation/delactylation of α-myosin heavy chain (α-MHC) at lysine 1897 determines its interaction with Titin. Reduced α-MHC K1897 lactylation has been observed in both mice and patients with heart failure. The K1897R mutation disrupts α-MHC–titin binding and exacerbates cardiac dysfunction, while sodium lactate supplementation or blockade of lactate efflux restores α-MHC K1897 lactylation and ameliorates heart failure [44]. In vascular smooth muscle cells (VSMCs), nuclear receptor subfamily 4 group A member 3 (NR4A3) was significantly upregulated in both mouse and human calcification specimens. It promotes lactate generation by directly binding to the promoter regions of ALDOA (aldolase, fructose-bisphosphate A) and PFKL (phosphofructokinase, liver type), which drives their transcription initiation. The increased lactate leads to elevated H3K18la levels and subsequent upregulation of PHOSPHO1, thereby accelerating arterial calcification [45]. Additionally, studies have found that TRAP1 mediates HDAC3-primed H4K12la to regulate VSMC senescence and macrophage polarization [46]. In parallel, TRAP1 suppresses HDAC3 activity, resulting in increased H4K12la that promotes vascular smooth muscle cell senescence by activating the SASP and exacerbating atherosclerosis. Together, these context-specific and cell-type-dependent functions of Kla underscore the potential of targeting lactylation as a therapeutic strategy for cardiovascular diseases, though clinical validation remains lacking.

The majority of findings stem from preclinical investigations, and there remains a critical lack of large-scale clinical validation linking Kla status to long-term cardiovascular outcomes in patients. Computational prediction is important for identifying functional lactylation sites and, consequently, for establishing mechanistic links between metabolic reprogramming and epigenetic remodeling in various cardiovascular diseases.

### 2.3. Kla and Neurological Disorders

Dysregulation of lactylation has been linked to the pathogenesis of neurological disorders, including neurodegenerative diseases [47,48] and neurodevelopmental disorders [47,49,50]. In psychiatric disorders, Hagihara et al. investigated chronic stress-induced depressive model mice and primary cultured mouse prefrontal cortex (PFC) neurons and observed that neuronal cells under chronic stress exhibited excessive lactate accumulation and alterations in Kla levels [26]. They also demonstrated the presence of Kla in PFC neurons of depressive model mice, and the Kla levels were negatively correlated with social behavior. The more susceptible the mice were to social stress and the higher their anxiety level, the higher the Kla levels in the PFC. These findings established the relationship between excessive neuronal activity under chronic stress, Kla levels in PFC neuronal cells, and the susceptibility to anxiety-like behaviors in mice [26]. In Alzheimer’s disease (AD), Wang et al. found that increased lactate levels in the brains of AD mice exposed to cigarette smoke (CS) could trigger the transcriptional activation of Nod-like receptor protein 3 (NLRP3) by promoting lactylation of lysine 12 on histone H4 (H4K12la), which subsequently led to mechanistic target of rapamycin (mTOR)-mediated autophagic dysfunction in microglia and exacerbated the pathological conditions of AD mice [48]. Moreover, lactate accumulation drives elevated H4K12la and H3K18la, exacerbating microglial overactivation, neuroinflammation, and amyloid-beta-associated pathology [51]. In Parkinson’s disease (PD), enhanced glycolysis and increased lactate promote H3K9la-mediated microglial polarization and dopaminergic neuronal loss [52]. Li et al. reported that Glis1 activated glycolysis to elevate lactate levels and enhanced H3K18la modification, which in turn upregulated pluripotency genes such as Oct4 and promoted the reprogramming of senescent mouse embryonic fibroblasts [47].

Although the pathological mechanisms of lactylation in neurological diseases are becoming increasingly clear, experimental approaches for identifying lactylation sites typically face challenges of long turnaround times, high costs, and limited throughput. This is particularly pronounced in neurodegenerative diseases, such as AD and PD, where pathogenic proteins (e.g., Tau, α-synuclein) possess complex structures and harbor multiple post-translational modifications, rendering the screening of key functional sites across the entire proteome using conventional mass spectrometry techniques extremely challenging. Therefore, the introduction of computational prediction algorithms represents a critical strategy for overcoming this bottleneck, enabling the efficient identification of potential modification sites, thereby substantially narrowing the scope of subsequent experimental validation and accelerating the mechanistic dissection of disease pathogenesis.

Due to the critical regulatory role of Kla in various major diseases, accurate and comprehensive identification of Kla sites is an essential prerequisite for elucidating the underlying molecular mechanisms of these diseases and uncovering novel therapeutic targets. This urgent research demand has substantially spurred the development of rapid, cost-effective computational strategies for Kla site prediction. In the following section, we provide an overview of representative published computational methods.

## 3. Data Resources and Computational Models for Kla Site Prediction

Recently, artificial intelligence (AI) and machine learning (ML) have advanced rapidly, providing powerful computational models for Kla site prediction. These models differ in aspects such as feature extraction, architectural design, and dataset construction, each presenting distinct advantages and limitations. We summarize the seven computational models for predicting Kla sites published from 2019 to 2025 (Table 1, Table 2 and Table 3).

### 3.1. Data Resources for Kla Site Prediction

Kla was first discovered in 2019, and there was no dedicated dataset available for its research at that time. Initially, researchers manually collected and integrated Kla-related data for further study and analysis. In 2021, Jiang et al. proposed FSL-Kla, the first Kla site prediction model [17]. From the latest literature, they manually collected 343 Kla sites across 191 proteins derived from Homo sapiens, Mus musculus, and Botryotinia fuckeliana. Every entry in the dataset is required to have a PubMed ID (PMID), with the exact position of Kla clearly defined. This rigorous data construction laid the foundation for subsequent research. Subsequently, Lv et al. obtained 1720 Kla sites in rice [53] and 273 Kla sites in Botrytis cinerea [54] from the literature [18]. In the Auto-Kla model developed by Lai and Gao [19], the researchers directly downloaded and utilized results from an independent experimental study [55]. The dataset incorporates 2375 Kla sites identified in gastric cancer AGS cells. In recent years, the data scale has achieved a leapfrog growth. Yang et al. [20] assembled a sample set consisting of 9560 human lactylation sites, all of which were identified through LC–MS/MS experiments [55,56,57,58]. In 2025, Ning et al. manually collected 23,984 known Kla sites across 7297 proteins from 14 eukaryotic and prokaryotic species reported in the literature to construct a comprehensive Kla benchmark dataset [21]. These species include Homo sapiens, Oryza sativa, Mus musculus, Rattus norvegicus, and others. Among all species, H. sapiens contributes the largest number, with 16,705 Kla sites in 4359 proteins. Compared to the datasets used in previous studies, such as Auto-Kla, DeepKla, and FSL-Kla, this dataset is substantially larger, showing an approximately 10-fold increase in scale. This surge in data scale marked the transition of Kla site prediction research from few-shot learning to a deep learning stage driven by big data (Table 2).

Despite the substantial increase in data volume, the benchmark dataset compiled by Ning et al. exhibits a pronounced species bias: 16,705 out of 23,984 Kla sites (approximately 69.6%) are derived from H. sapiens, while other species contribute far fewer sites. Such an imbalance could potentially bias the trained model toward human-specific sequence patterns [21]. Future collection of more non-human Kla sites (e.g., from plants, fungi, or non-mammalian animals) will further reduce species bias and enhance model robustness.

### 3.2. Computational Models for Kla Site Prediction

Known computational methods for Kla site prediction are presented below. However, direct performance comparisons across studies are complicated by differences in datasets, species composition, peptide lengths, class-balancing strategies, and validation protocols. The models summarized below should be understood within each study’s experimental context and not treated as directly comparable model rankings.

#### 3.2.1. FSL-Kla

FSL-Kla is the first computational model for Kla site prediction, developed by Jiang et al. in 2021 [17]. The model integrates few-shot learning with a multi-feature hybrid ensemble deep learning architecture to address the challenge of limited Kla sites data [17]. For balancing datasets and reducing overfitting, two feature sets are used: set 1 (AAC, CTriad, AAindex, DPC, CTDT, CTDC, and CKSAAP) and set 2 (ASA, BTA, PSSM, and SS). For set 1, FSL-1 applies SMOTE for data enrichment and removes Tomek links. For set 2 (non-continuous features), FSL-2 uses random undersampling (RUS). Subsequently, EDL-1 (cascade ensemble with EasyEnsemble) processes set 1, while EDL-2 (parallel ensemble with AdaBoost) handles set 2. The raw outputs of EDL-1 and EDL-2 are first calibrated using Platt’s logistic model. Subsequently, these calibrated probabilities are stacked via a refined penalized logistic regression (PLR) method to construct the multi-feature hybrid system (mFHS). The model was rigorously validated using stratified cross-validation, ultimately attaining a final AUC of 0.889. FSL-Kla exhibits excellent robustness and strong interpretability in small-sample Kla prediction scenarios. Nevertheless, it relies heavily on manually crafted features, which restricts its capability to mine implicit high-order sequence patterns and weakens its generalization ability on diverse independent datasets.

#### 3.2.2. DeepKla

DeepKla is the first computational framework for predicting Kla sites in rice, proposed by Lv et al. in 2022 [18]. This model integrates four tightly coupled components: a word embedding layer, a convolutional neural network (CNN), bidirectional gated recurrent units (BiGRU), and an attention mechanism layer. To avoid biased features resulting from artificial design, the embedding layer only takes protein sequences as input and automatically extracts sequence features. The architecture leverages CNN for sequence encoding, while BiGRU captures long-range information from protein sequences, and the attention mechanism pinpoints key positional information. They evaluated the model using 5-fold cross-validation and an independent test set. The architecture with the attention mechanism achieved higher accuracy (94.07%). In contrast, the BiLSTM-based architecture yielded lower accuracy (85.59%). These results indicate that BiGRU is more advantageous for predicting Kla sites. The model achieved an AUC of 0.9901 under 5-fold cross-validation on the training dataset and an AUC of 0.9722 on the independent test set, demonstrating the robustness of DeepKla in identifying Kla and non-Kla sites and excellent prediction ability and transferability. The main advantages of DeepKla are automatic feature learning and avoidance of manual feature bias. Nevertheless, as a data-driven deep learning model, it requires sufficient labeled data for training and has relatively low model interpretability compared with traditional feature-based methods.

#### 3.2.3. Auto-Kla

Auto-Kla, developed by Lai and Gao in 2023, discriminates Kla sites using automated machine learning (AutoML), addressing the challenge of overfitting/underfitting on small datasets and complex manual hyperparameter tuning [19]. Built on the AutoGluon tool, the model uses the ELECTRA discriminator structure proposed by Google as its core, comprising an adaptive embedding module, a transformer encoder module, and a multi-layer perceptron classifier module. This architecture allows the system to automatically leverage optimal techniques when appropriate. Tasks such as hyperparameter tuning, model selection and combination, architecture search, and data processing are handled automatically. Meanwhile, the adaptive embedding module extracts token-level and position-level features for each residue, thereby circumventing the limitations of manual feature selection. Validated by 10-fold cross-validation, Auto-Kla outperforms prior models in gastric cancer Kla site prediction, achieving an average AUROC of 92.34% (±0.62%), which is 4.61% higher than DeepKla. The model exhibits a smaller standard deviation of all evaluation indicators, demonstrating better prediction performance, generalization ability, robustness, and stability than DeepKla. Auto-Kla offers key advantages, including automated model construction via AutoML, eliminating manual feature engineering and hyperparameter tuning, as well as high stability, robustness, and strong generalization across PTM types. Nevertheless, the method has several drawbacks: the study does not explore advanced sampling strategies for imbalanced data; it leverages only primary sequence information while neglecting structural or physicochemical features; and the Transformer architecture offers poor biological interpretability.

#### 3.2.4. ABFF-Kla and EBFF-Kla

ABFF-Kla (attention-based feature fusion Kla model) and EBFF-Kla (embedding-based feature fusion Kla model), developed by Yang et al. in 2024, are human Kla site prediction models that use natural language processing techniques to automatically extract and fuse 3D structural features with sequence features [20]. This overcomes the limitations of previous models, which relied solely on primary sequence features or manually designed structural features. The authors used the protein structure-generated residue contact map to select the nearest contacting residues around the target lysine site. Validation results indicated that combining protein sequence features and spatial structure features leads to better predictive performance than using sequence features alone. This conclusion was validated through comparisons with existing sequence-only models such as DeepKla and Auto-Kla. Specifically, ABFF-Kla (LSTM) has the highest performance, followed by EBFF-Kla (Bi-GRU). The two ABFF-Kla models exhibit higher specificity and sensitivity than the others, indicating that the attention-based feature fusion framework can supply richer information than the embedding-based method, leading to better predictive performance. The major strengths include automatic 3D feature extraction, effective integration of sequence and structure, and superior predictive power. However, the models depend on complete protein 3D structures, which are not universally available; they also lose partial spatial information by projecting 3D contacts into 2D segments, and their complex deep learning architecture reduces biological interpretability.

#### 3.2.5. HybridKla

In 2025, Ning et al. leveraged the hybrid encoding system together with deep learning to develop a prediction tool named HybridKla [21]. They manually curated the largest benchmark dataset to date, comprising 23,984 experimentally verified Kla sites across 7297 proteins from 14 species, and developed a hybrid system that incorporated eight complementary strategies for feature encoding. This system synergistically combines two automated encoders (a fine-tuned protein language model (ESM2) and a six-layer long short-term memory (LSTM) module) with a composition-based framework that consists of six handcrafted descriptors, namely ACF, AAINDEX, CKSAAP, GPS, OBC, and PseAAC. They developed base models through the integration of deep neural networks (DNNs) and the associated feature extraction approaches. In this model, Ning et al. performed ablation experiments to test each feature’s contribution and used leave-one-out validation to confirm cross-species robustness [21]. The results show that on the same benchmark dataset, HybridKla achieved an AUC of 0.8460, which is markedly higher than that of Auto-Kla (0.6563) and DeepKla (0.5563), corresponding to improvements of 28.90% and 52.07%, respectively, demonstrating significant performance gains over existing tools. Although the multi-feature hybrid architecture of HybridKla achieves strong predictive performance, it inevitably increases computational cost and model complexity. Its performance is highly dependent on the large-scale training dataset, and its generalization to poorly studied or non-model species has not been fully validated. Furthermore, despite the use of attention weight visualization and t-SNE feature projection, the interpretability of its site-specific prediction decisions remains limited.

#### 3.2.6. PCBert-Kla

PCBert-Kla is a feature-fusion deep learning model based on ProtBert [22]. This model leverages ProtBert (a protein-specific BERT model) pretrained on UniRef100, which contains 217 million sequences. Each sequence is regarded as a separate document, and the model does not perform next-sentence prediction. ProtBert generates 1024-dimensional feature vectors that effectively capture global and local contextual information from protein sequences. The model combines diverse physicochemical properties, including molecular weight, isoelectric point, amino acid composition, secondary structure content, hydrophobicity, and net charge. This combined feature space avoids the limitations of sequence-only representations. An attention mechanism in the fully connected layers automates the feature selection process. They preserved only the first four BertEncoder layers during training (discarding the rest) and, rather than freezing them, fine-tuned the entire architecture. This study directly reused the benchmark dataset established by Lv et al. for DeepKla, without any modifications, to ensure a fair comparison [18]. Zhang et al. performed ablation experiments to validate the contribution of physicochemical properties and the attention mechanism and used independent testing to confirm the model’s generalization capability [22]. The results show that PCBert-Kla achieved an AUC of 0.9728 on the independent set, demonstrating significant performance gains over existing tools such as DeepKla and Auto-Kla. The model fuses deep sequence features with physicochemical properties and uses attention in fully connected layers to dynamically weight features, improving accuracy and robustness. However, its decision-making is hard to interpret, and performance relies heavily on the ProtBert pre-trained model.

## 4. Challenges and Perspectives

Kla, a novel post-translational modification formally reported in 2019, regulates the functions of both histones and non-histone proteins through enzymatic or non-enzymatic mechanisms [3,4]. This modification plays a pivotal role in diverse physiological and pathological processes. Accurate identification of Kla sites is fundamental to deciphering the molecular mechanisms and disease associations. However, conventional mass spectrometry-based approaches are inherently limited by time-consuming procedures, high costs, and restricted throughput, rendering them inadequate for proteome-wide screening. Against this backdrop, computational prediction methods have rapidly emerged, evolving from few-shot learning to large-scale, data-driven frameworks and from single sequence features to multi-source information integration, thereby providing critical support for Kla site prediction.

### 4.1. Dataset Limitations and Bias

Despite the rapid expansion of data scale, several intrinsic limitations warrant careful consideration. Class imbalance remains a persistent challenge. Early datasets like FSL-Kla exhibited an extreme ratio of approximately 1:20, biasing models toward the majority class. While later models adopted 1:1 balancing, such artificial equilibrium fails to mirror the true biological scarcity of Kla sites in vivo, potentially leading to over-optimistic performance estimates. Species bias is also evident: while HybridKla expanded the scope to 14 species, the majority of data (>69%) still originates from Homo sapiens, limiting the transferability of predictors to non-human organisms. Furthermore, heterogeneity in data processing—ranging from peptide window sizes (21–51 residues) to sequence redundancy thresholds (CD-HIT 30–70%)—complicates rigorous cross-model comparison. Finally, an experimental validation bias exists, as most positive samples derive from MS-biased cell lines (e.g., gastric cancer AGS cells in Auto-Kla), while negative samples are often computationally inferred rather than experimentally confirmed. Future efforts should prioritize the integration of structural biology and language models to construct more standardized, multi-species benchmarks that better reflect the true complexity of the lactylome.

### 4.2. Evolution of Prediction Models

**Figure 2:**
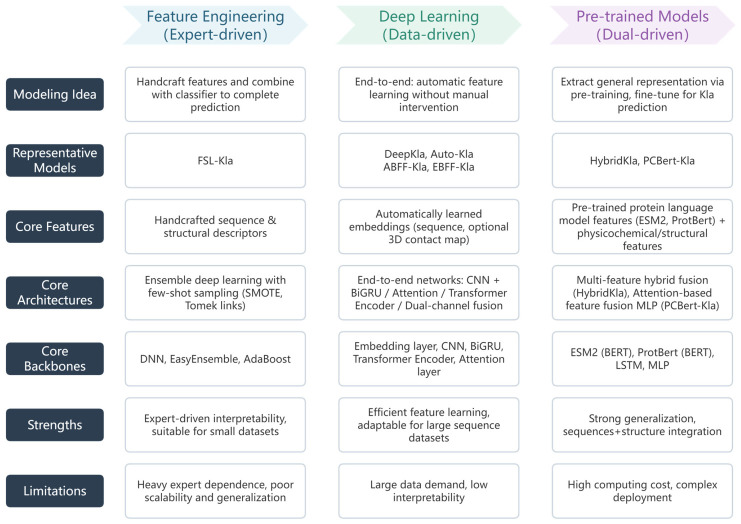
Technical evolution and comparison of lysine lactylation site prediction paradigms. This figure compares three major methodological paradigms for Kla site prediction: expert-driven feature engineering, data-driven deep learning, and dual-driven pre-trained models. Columns represent paradigms, while rows present a comparison of core modeling ideas, representative models, features, architectures, strengths, and limitations. The left-to-right progression shows the shift from handcrafted features to modern pre-trained language model frameworks.

The development history of the Kla site prediction technology is essentially an evolution of feature extraction methods from “manual design” to “automatic learning” and an upgrade of model architectures from “single sequence” to “multi-model fusion”, which directly drives the stepwise leap in prediction performance and generalization ability (Figure 2).

In the early stage, with only 343 non-redundant Kla sites, FSL-Kla combined eight sequence-based and three structure-based features, applied SMOTE and Tomek links, and used ensemble deep learning. It achieved an AUC of 0.889, proving that refined manual features are key to generalization in data-scarce scenarios. With the accumulation of data, DeepKla and Auto-Kla initiated the era of end-to-end automatic feature extraction. DeepKla pioneered an end-to-end architecture (embedding + CNN + BiGRU + attention) to predict rice Kla sites, achieving an AUC of 0.9722. Auto-Kla introduced the concept of Automated Machine Learning (AutoML) with a Transformer-based model, reaching an AUC of 0.9234 on the gastric cancer dataset, demonstrating stronger robustness than DeepKla. However, a critical distinction must be made between performance on original training datasets and true cross-species generalization: when the published Auto-Kla and DeepKla models were directly applied to a comprehensive multi-species benchmark dataset (14 species, 23,984 experimentally verified Kla sites), DeepKla’s AUC dropped to 0.5563 and Auto-Kla’s to 0.6563, exposing the generalization bottleneck of models trained on limited, species-specific datasets in handling heterogeneous biological data. This stark contrast underscores that excellent performance on a single closed dataset does not guarantee transferability to unrepresented species or varied biological contexts—an essential consideration for practical use.

Recently, the era of multi-model fusion and pre-trained models has emerged, characterized by two complementary approaches: (i) integration of 3D structural information and (ii) application of pre-trained language models (PLMs). ABFF-Kla and EBFF-Kla took the lead in converting protein three-dimensional contact maps predicted by AlphaFold into features, designing dual-stream fusion architectures (attention-based fusion or early fusion). HybridKla integrated ESM2, LSTM, and handcrafted features, achieving an AUC of 0.8460 on the benchmark dataset, with stable cross-species validation (e.g., AUC of 0.8325 on mouse data), demonstrating excellent cross-species generalization ability. PCBert-Kla utilized ProtBert to extract deep semantic embeddings combined with physicochemical properties, reaching an accuracy of 0.9497 on the independent test set. Notwithstanding the above-mentioned differences in experimental setups, the continual integration of sequence, structural, and semantic information from large-scale pre-trained models is clearly driving substantial breakthroughs in both the accuracy and cross-scenario transferability of Kla site prediction models.

### 4.3. Model Interpretability

Alongside performance improvements, model interpretability and feature importance analysis have become essential for revealing the biological basis of Kla. PCBert-Kla evaluated the contribution of multiple physicochemical descriptors and identified net charge as the most influential feature, followed by isoelectric point, helix content, and amino acid composition. HybridKla conducted ablation experiments on eight combined feature types and confirmed that ESM2 protein language model features, LSTM-based temporal features, and CKSAAP represent the top three contributors to prediction performance. ABFF-Kla and EBFF-Kla further demonstrated that three-dimensional structural features derived from residue contact maps provide critical complementary information that cannot be captured by sequence alone. DeepKla and Auto-Kla employed attention mechanisms and t-SNE visualization to illustrate that models effectively capture local sequence patterns around target lysine sites. FSL-Kla evaluated 11 manually designed features and found amino acid composition and secondary structure to be the most predictive. Collectively, these studies demonstrate that sequence context, physicochemical properties, spatial structure, and pre-trained semantic embeddings act synergistically to enable accurate Kla site recognition.

### 4.4. Expanding Future Trends

As experimental technologies continue to advance and computational methodologies evolve, Kla site prediction will advance toward enhanced precision, scalability, and interpretability. Specifically, future developments may focus on the following directions:

(1) Negative sample selection strategies. During the training process, computational methods typically require negative samples. However, in the context of PTM site prediction, experimentally validated negative sites are extremely scarce or entirely absent. To address this, the majority of existing studies resort to randomly sampling negative instances from unlabeled data. Critically, unlabeled lysines cannot be assumed to be true negatives—many may in fact harbor genuine modification sites that have not yet been identified. Training with such unreliable negatives introduces label noise, which can inflate model performance estimates and bias feature learning, ultimately undermining the prediction accuracy of Kla models. Specifically, negative samples contaminated by hidden positive sites further impair model reliability. Consequently, considerable opportunities remain for designing more rational negative sample selection strategies. Promising future directions include the adoption of positive-unlabeled (PU) learning frameworks that circumvent the need for explicit negative sets, the hard-negative mining techniques that iteratively select the most informative negative candidates, and the construction of experimentally curated negative datasets to provide a more trustworthy foundation for both model training and evaluation.
(2) Model interpretability. Interpretability is another significant challenge facing deep learning methods. In bioinformatics, good interpretability not only helps to assess model performance but also enables researchers to better understand the underlying biomolecular mechanisms. Future efforts should focus on designing methods that interpret and visualize complex relationships, transforming the “black box” of computation into a biologically interpretable “white box”.
(3) Integration of computational and biological experiments. The integration of computational and biological experiments is equally crucial. Computational scientists tend to focus more on algorithm-level performance evaluation while neglecting the necessity of biological validation. Due to a lack of substantial cooperation with biologists, prediction results are difficult to verify experimentally, thus failing to achieve the goal of reducing costs and improving efficiency through computational methods. Therefore, future studies will benefit from close collaboration between computational scientists and biologists to deepen our understanding of Kla’s biological roles.

## Figures and Tables

**Table 1:** Summary of the lysine lactylation site prediction models.

Model Name	Brief Introduction	Web Server URL	GitHub/Source Code URL
FSL-Kla ^1^	FSL-Kla is a few-shot learning-based multi-feature hybrid system that integrates 8 sequence-based and 3 structure-based features to address the small dataset problem through heterogeneous few-shot strategies and ensemble methods.	http://kla.zbiolab.cn/ (accessible on 24 May 2026)	Not publicly available
DeepKla	DeepKla is a deep learning-based predictor originally developed for lysine lactylation sites in rice, utilizing a hybrid architecture of a supervised embedding layer, CNN, bidirectional GRU networks, and an attention mechanism.	http://lin-group.cn/server/DeepKla (accessible on 24 May 2026)	https://github.com/linDing-group/DeepKla (accessible on 24 May 2026)
Auto-Kla ^2^	Auto-Kla is an AutoML-based transformer model that leverages an embedding layer and a multi-head self-attention mechanism to discriminate lysine lactylation sites in gastric cancer cells.	http://tubic.org/Kla (accessible on 24 May 2026)	https://github.com/tubic/Auto-Kla (accessible on 24 May 2026)
ABFF-Kla ^3^	ABFF-Kla is an attention-based feature fusion deep learning framework that integrates protein sequence and 3D structural features through the attention layer to predict human lysine lactylation sites.	No dedicated web server provided	https://github.com/ispotato/Lactylation_model (accessible on 24 May 2026)
EBFF-Kla ^3^	EBFF-Kla is an embedding-based feature fusion deep learning framework that integrates protein sequence and 3D structural features through the embedding layer to predict human lysine lactylation sites.	No dedicated web server provided	https://github.com/ispotato/Lactylation_model (accessible on 24 May 2026)
HybridKla	HybridKla is a multi-feature hybrid deep learning framework that integrates automated encodings from a protein language model with composition-based handcrafted sequence descriptors to predict lysine lactylation sites.	http://transkla.zzu.edu.cn/ (accessible on 24 May 2026)	https://github.com/kongxianzw/HybridKla/ (accessible on 24 May 2026)
PCBert-Kla	PCBert-Kla is a feature-fusion deep learning model based on ProtBert that integrates physicochemical properties with an attention mechanism in the fully connected layer to identify lysine lactylation sites.	http://pcbert-kla.lin-group.cn/ (accessible on 24 May 2026)	https://github.com/ZhangHongqi215/PCBert-Kla (accessible on 24 May 2026)

^1^ This web server no longer offers lysine lactylation site prediction and has been repurposed for unrelated use. ^2^ Website name changed to Auto-PTM; original prediction tool remains fully available at this URL. ^3^ ABFF-Kla and EBFF-Kla are two models from the same paper (Yang et al.) [20].

**Table 2:** Characteristics of benchmark datasets for lysine lactylation site prediction models.

Model	Pos (Train)	Neg (Train)	Balancing Strategy	Species	Peptide Length	Redundancy Filter	Independent Test (Pos/Neg)
FSL-Kla	343	≈6860	SMOTE + Tomek links/RUS ^1^	3	21 (±10)	Peptide-level	Cross-validation ^2^
DeepKla	1720	1767	Oversampling	2 ^3^	51 (±25)	CD-HIT (30%)	177/177
Auto-Kla	1912	19,562	Oversampling (10×) ^4^	1	51 (±25)	CD-HIT (70%)	≈382/≈3912
ABFF-Kla/EBFF-Kla ^5^	9560	9560	Random under-sampling	1	31–39 ^6^	CD-HIT (70%)	755/755
HybridKla	23,984	23,984	Random under-sampling	14 ^7^	51 (±25)	Peptide-level	1672/1672
PCBert-Kla ^8^	1720	1767	Oversampling	2 ^3^	51 (±25)	CD-HIT (30%)	177/177

^1^ FSL-Kla applied SMOTE + Tomek links for feature set 1 and random under-sampling (RUS) for feature set 2. ^2^ Performance was evaluated by 4-, 6-, 8-, and 10-fold cross-validation; no independent test set was constructed. ^3^ Trained on Oryza sativa, tested on Botrytis cinerea. ^4^ Positive samples were oversampled 10-fold within each cross-validation training fold to achieve a 1:1 ratio. ^5^ These two models are presented together because they share the same dataset, evaluation pipeline, and overall objective, differing only in how they fuse sequence and structural information. These two models will also be considered together in the following sections for further comparison and analysis. ^6^ Multiple peptide lengths (31, 35, 39) were evaluated; a length of 35 was found to be optimal. ^7^ Includes 14 species, predominantly Homo sapiens (16,705 sites). ^8^ PCBert-Kla directly used DeepKla’s benchmark dataset.

**Table 3:** Comparison of validation strategies and core performance metrics.

Model Name	Validation Method	Performance (Core Metrics) ^1^
FSL-Kla	4/6/8/10-fold stratified cross-validation. Probability calibration with Brier score evaluation. Comparison with conventional machine learning methods/single-feature-based models.	Mean AUC = 0.889. Brier score = 0.0181. Outperformed conventional machine learning methods by 11.7% AUC. Outperformed the best single-feature model by 16.2% AUC.
DeepKla	5-fold cross-validation (rice). Independent test set (Botrytis cinerea). Ablation studies.	Training set: AUC = 0.9901, ACC = 0.9949, and MCC = 0.8789. Independent test set: AUC = 0.9722, ACC = 0.9322, and MCC = 0.8671. Ablation studies showed the attention mechanism improved accuracy by 1.98%, and the BiGRU module outperformed BiLSTM by 8.48% in accuracy.
Auto-Kla	10-fold cross-validation. Independent test set. Standard deviation. Comparison with DeepKla.	Independent test set: AUC = 92.34 ± 0.62%, ACC = 91.21 ± 1.58%, and MCC = 55.40 ± 2.33%. Auto-Kla exhibited smaller standard deviations, demonstrating stronger generalization ability and stability against different data partitions. Outperformed DeepKla by 4.61% AUC, 1.41% ACC, and 11.2% MCC.
ABFF-Kla/EBFF-Kla	10-fold cross-validation. Independent validation set. Comparison with DeepKla/Auto-Kla.	Independent validation set: (1) ABFF-Kla: AUC = 0.9399, ACC = 0.8300, and MCC = 0.6829. (2) EBFF-Kla: AUC = 0.9332, ACC = 0.8136, and MCC = 0.6594. Both models outperformed DeepKla and Auto-Kla across all metrics.
HybridKla	5-fold cross-validation. Leave-one-species-out validation. Independent test set. Ablation studies. Comparison with DeepKla/Auto-Kla.	Overall AUC = 0.8460 and AUPRC = 0.8202. Independent test set: AUC = 0.8325 and AUPRC = 0.8018. Leave-one-species-out validation indicates strong cross-species generalization. Ablation studies confirmed the synergistic effect of ESM2, LSTM, and CKSAAP features. Outperformed DeepKla by 52.07% AUC. Outperformed Auto-Kla by 28.90% AUC.
PCBert-Kla	5-fold cross-validation. Independent external test set. Ablation studies. Comparison with DeepKla/Auto-Kla.	5-fold CV: AUC = 0.9953, ACC = 0.9837, and MCC = 0.9677. Independent external test set: AUC = 0.9728, ACC = 0.9497, and MCC = 0.8999. Ablation studies demonstrate that the attention mechanism enhancement in the fully connected layer effectively boosts model performance. Outperformed DeepKla by 0.29% AUC, 1.75% ACC, and 3.78% MCC. Outperformed Auto-Kla by 16.21% AUC, 26.04% ACC, and 51.78% MCC.

^1^ Models differ in training data, species, balancing strategies, and validation methods; thus, absolute AUC values are not directly comparable.

## Data Availability

No new data were generated or analyzed in this study. Data sharing is not applicable to this article.
